# Decline in Diarrhea Mortality and Admissions after Routine Childhood Rotavirus Immunization in Brazil: A Time-Series Analysis

**DOI:** 10.1371/journal.pmed.1001024

**Published:** 2011-04-19

**Authors:** Greice Madeleine Ikeda do Carmo, Catherine Yen, Jennifer Cortes, Alessandra Araújo Siqueira, Wanderson Kleber de Oliveira, Juan José Cortez-Escalante, Ben Lopman, Brendan Flannery, Lucia Helena de Oliveira, Eduardo Hage Carmo, Manish Patel

**Affiliations:** 1 Secretariat for Health Surveillance (Secretaria de Vigilância em Saúde), Ministry of Health, Brasilia, Brazil; 2 National Center for Immunization and Respiratory Diseases, Centers for Disease Control and Prevention, Atlanta, Georgia, United States of America; 3 Epidemic Intelligence Service, Centers for Disease Control and Prevention, Atlanta, Georgia, United States of America; 4 General Coordination for Analysis of Epidemiologic Information (Coordenação-Geral de Informações e Análises Epidemiológicas), Department for Analysis of Health Data (Departamento de Análise de Situação de Saúde), Secretariat for Health Surveillance, Ministry of Health, Brasilia, Brazil; 5 Pan American Health Organization, Brasilia, Brazil; 6 Pan American Health Organization, Washington, District of Columbia, United States of America; Menzies School of Health Research, Australia

## Abstract

**Background:**

In 2006, Brazil began routine immunization of infants <15 wk of age with a single-strain rotavirus vaccine. We evaluated whether the rotavirus vaccination program was associated with declines in childhood diarrhea deaths and hospital admissions by monitoring disease trends before and after vaccine introduction in all five regions of Brazil with varying disease burden and distinct socioeconomic and health indicators.

**Methods and Findings:**

National data were analyzed with an interrupted time-series analysis that used diarrhea-related mortality or hospitalization rates as the main outcomes. Monthly mortality and admission rates estimated for the years after rotavirus vaccination (2007–2009) were compared with expected rates calculated from pre-vaccine years (2002–2005), adjusting for secular and seasonal trends. During the three years following rotavirus vaccination in Brazil, rates for diarrhea-related mortality and admissions among children <5 y of age were 22% (95% confidence interval 6%–44%) and 17% (95% confidence interval 5%–27%) lower than expected, respectively. A cumulative total of ∼1,500 fewer diarrhea deaths and 130,000 fewer admissions were observed among children <5 y during the three years after rotavirus vaccination. The largest reductions in deaths (22%–28%) and admissions (21%–25%) were among children younger than 2 y, who had the highest rates of vaccination. In contrast, lower reductions in deaths (4%) and admissions (7%) were noted among children two years of age and older, who were not age-eligible for vaccination during the study period.

**Conclusions:**

After the introduction of rotavirus vaccination for infants, significant declines for three full years were observed in under-5-y diarrhea-related mortality and hospital admissions for diarrhea in Brazil. The largest reductions in diarrhea-related mortality and hospital admissions for diarrhea were among children younger than 2 y, who were eligible for vaccination as infants, which suggests that the reduced diarrhea burden in this age group was associated with introduction of the rotavirus vaccine. These real-world data are consistent with evidence obtained from clinical trials and strengthen the evidence base for the introduction of rotavirus vaccination as an effective measure for controlling severe and fatal childhood diarrhea.

*Please see later in the article for the Editors' Summary*

## Abstract 2

A time series analysis by Manish Patel and colleagues shows that the introduction of rotavirus vaccination in Brazil is associated with reduced diarrhea-related deaths and hospital admissions in children under 5 years of age.

## Editors' Summary

**Background:**

Diarrheal disease, usually caused by infectious agents, is the second major cause of death in children aged under five years. As highlighted in a recent *PLoS Medicine* series, access to clean water and improved sanitation is the key to the primary prevention of diarrheal illnesses. Yet despite the targets of Millennium Development Goal 7 to half the number of people without access to clean water or improved sanitation by 2015, over one billion people worldwide do not currently have access to clean water and over two billion do not currently have access to improved sanitation.

Since enteric viruses are primarily transmitted directly from one person to another, they cannot be controlled completely by improvements in sanitation. Therefore, although not replacing the urgent need to provide access to clean water and improved sanitation for all, vaccination programs that protect young children against some infections that cause diarrhea, such as rotavirus, which accounts for one-third of all child deaths caused by diarrhea, are a pragmatic way forward. As large clinical trials have shown the safety and efficacy of rotavirus vaccines in population settings, in July 2009, the World Health Organization recommended including rotavirus vaccines into every country's national immunization programs.

**Why Was This Study Done?:**

Although the protective effect of rotavirus vaccines has been assessed in various high-, middle-, and low-income settings, for reasons that remain unclear, the efficacy of live, oral rotavirus vaccines appears to be dependent on geographical location and correlated to the socioeconomic status of the population. Because of these concerns, evaluating the health impact of large-scale rotavirus vaccine programs and ensuring their equity in a real-world setting (rather than in clinical trial conditions) is important.

Therefore, the researchers addressed this issue by conducting this study to evaluate the effect of rotavirus vaccination on mortality and hospital admissions for diarrhea due to all causes among young children in the five regions of Brazil. The researchers chose to do this study in Brazil because of the high incidence of diarrhea-related deaths and hospital admissions and because five years ago, in July 2006, the Brazilian Ministry of Health introduced the single-strain rotavirus vaccine simultaneously in all 27 states through its national immunization program—allowing for “before” and “after” intervention analysis.

**What Did the Researchers Do and Find?:**

The researchers obtained data on diarrheal deaths and hospital admissions in children aged under five years for the period 2002–2005 and 2007–2009 and data on rotavirus vaccination rates. The researchers got the data on diarrhea deaths from the Brazilian Mortality Information System—the national database of information collected from death certificates that covers 90% of all deaths in Brazil. The data on hospital admissions came from the electronic Hospital Information System of Brazil's Unified Health System (Sistema Unico de Saúde, SUS)—the publicly funded health-care system that covers roughly 70% of the hospitalizations and includes information on all admissions (from public hospitals and some private hospitals) authorized for payment by the Unified Health System. The researchers got regional rotavirus vaccination coverage estimates for 2007–2009 from the information department of the Ministry of Health, and estimated coverage of the two doses of oral rotavirus vaccine by taking the annual number of second doses administered divided by the number of infants in the region.

In 2007, an estimated 80% of infants received two doses of rotavirus vaccine, and by 2009, this proportion rose to 84% of children younger than one year of age. The researchers found that in the three years following the introduction of rotavirus vaccination, diarrhea-related mortality rates and admissions among children aged under five years were respectively 22% and 17% lower than expected, with a cumulative total of 1,500 fewer diarrhea deaths and 130,000 fewer admissions. Furthermore, the largest reductions in deaths and admissions were among children who had the highest rates of vaccination (less than two years of age), and the lowest reductions were among children who were not eligible for vaccination during the study period (aged 2–4 years).

**What Do These Findings Mean?:**

These findings suggest that the introduction of rotavirus vaccination in all areas of Brazil is associated with reduced diarrhea-related deaths and hospital admissions in children aged under five years. These real-world impact data are consistent with the clinical trials and strengthen the evidence base for rotavirus vaccination as an effective measure for controlling severe and fatal childhood diarrhea.

These findings have important global policy implications. In middle-income countries, such as Brazil, that are not eligible for financial support from donors, the potential reductions in admissions and other health-care costs will be important for cost-effectiveness considerations to justify the purchase of these still relatively expensive vaccines.

**Additional Information:**

Please access these Web sites via the online version of this summary at http://dx.doi.org/10.1371/journal.pmed.1001024

- *PLoS Medicine* has published a series on water and sanitation
- More information is available from the World Health Organization on diarrheal illness in children
- More information is available about rotavirus vaccines from the World Health Organization, the US Centers for Disease Control and Prevention, and the Rotavirus Vaccine Program

## Introduction

In July 2009, the World Health Organization recommended introduction of rotavirus vaccines into national immunization programs of all countries worldwide for controlling severe rotavirus disease, which accounts for approximately one-third of the 1.34 million diarrhea deaths and 9 million hospital admissions worldwide among children younger than 5 y of age [1]–[3]. Evaluating the health impact of large-scale programs, and ensuring their equity, is considered a top priority in global health for well-informed policy decisions [4]–[6]. This is particularly important for rotavirus vaccine programs because challenges remain concerning performance of the vaccine in regions with the highest morbidity and mortality [7],[8].

The protective effect of rotavirus vaccines has been assessed in various high-, middle-, and low-income settings. For unclear reasons, efficacy of live, oral rotavirus vaccines is location-dependent, with a gradient in immune response and protective efficacy that correlates with the socioeconomic status of the population [7],[8]. In a clinical trial from Latin America, the single-strain human rotavirus vaccine Rotarix (GlaxoSmithKline Biologicals) showed a protective efficacy of 85% against severe rotavirus disease (i.e., hospital admissions) [9],[10]. Efficacy was lower in the middle-income settings of South Africa (77%) and El Salvador (76%) and the low-income setting of Malawi (49%) [11],[12]. A similar gradient in efficacy has also been shown for the pentavalent rotavirus vaccine RotaTeq (Merck) in clinical trials [13]–[15] and post-licensure evaluations [16]–[18].

Although rotavirus vaccines have only recently been introduced, the population-level effect of vaccination has been assessed in various settings. A large reduction in national diarrhea hospital admissions after the introduction of pentavalent rotavirus vaccine has been shown in high-income settings [19]–[22]. While these data are promising, evaluations from middle- and low-income settings, most of which are using the single-strain vaccine [23], are limited. A recent study from Mexico demonstrated significant reductions in infant diarrhea mortality early after rotavirus vaccine introduction [24]. Early evaluations of the impact of rotavirus vaccine in Brazil have shown a reduction in rotavirus and diarrhea hospitalizations during the first year after vaccine introduction [25]–[27]. However, Brazil is a large country with heterogeneous socioeconomic conditions and a high burden of diarrheal disease, where diarrhea deaths and admissions occur year-round and disease trends had been declining in recent years before vaccination [28]. In such settings, accounting for secular declines and monitoring diarrhea trends for a longer duration after vaccine introduction are necessary to isolate the effect of vaccine from other concurrently implemented interventions or changes in practice. Thus, we conducted a comprehensive national evaluation quantifying the sustained effect of a vaccination program in Brazil on both relevant outcomes, diarrhea deaths and diarrhea admissions from all causes, using an interrupted time-series analysis.

In 2006, the Brazilian Ministry of Health introduced the single-strain rotavirus vaccine, Rotarix, simultaneously in all 27 states through its national immunization program in order to accelerate reaching the fourth Millennium Development Goal of reduced child mortality. The primary objective of this study was to evaluate the effect of rotavirus vaccination by analyzing trends in mortality and hospital admissions for diarrhea due to all causes among young children in the five regions of Brazil.

## Methods

### Ethics Statement

This evaluation involved analysis of existing, publicly available datasets. Because these are publicly available non-identifiable data, the Brazilian Ministry of Health and the United States Centers for Disease Control and Prevention human subjects' offices deemed that an ethics statement was not required for this work.

### Setting and Design

Brazil is a middle-income Latin American country with an annual birth cohort of ∼3 million infants and a gross domestic product per capita of US$8,300. Brazil's 27 states are divided into five geographic regions (North, Northeast, Southeast, South, and Central-West) with distinct socioeconomic and health indicators (Table 1). The United Nations Development Program classifies countries with a human development index (HDI) below 0.90 as “developing” [29]. In 2005, among 182 countries ranked in decreasing order of HDI, Brazil overall (HDI  = 0.805) ranked 75^th^ (for general comparison, Mexico [HDI = 0.844] ranked 53^rd^ and the United Kingdom ranked 21^st^ [HDI  = 0.947]). The Northeast region of Brazil individually had an index (HDI = 0.720) below that of Bolivia (HDI = 0.722), a developing country that ranked 113^th^.

**Table 1:** Basic demographic, socioeconomic, and health indicators in Brazil, by region.

Region	HDI^a^	Population <1 y, 2009^b^	<5-y Mortality per 1,000 Live Births, 2005^c^	Percent Deaths <5 y Due to Diarrhea, 2005^c^	Rotavirus Vaccine Coverage among Children <1 y^d^	
					2008	2009
All regions	0.805	3,013,689	25.4	4.1	81.3	84.3
Northeast	0.72	1,005,387	37.3	6.5	79.0	82.4
North	0.764	309,789	27.6	6.2	64.6	69.5
Central-West	0.815	232,233	21.2	3.5	85.1	89.4
Southeast	0.824	1,119,725	17.9	1.7	86.1	87.9
South	0.829	346,555	16.1	1.7	84.6	87.3

a The HDI is a summary index that ranges from 0 (lowest) to 1 (highest), composed of life expectancy at birth, adult literacy rate, school enrollment rate, and per capita gross domestic product. The index is produced by the United Nations Development Program. HDIs for Brazilian regions were last published in 2005 [29].

b Source: Ministry of Health, Brasilia, Brazil [33].

c Health Indicator and Basic Data in Brazil (IDB) [32]. The <5-y mortality per 1,000 live births denotes the probability of dying between age 0 and 5 y per 1,000 live births.

d Vaccination coverage estimated based on number of second doses of rotavirus vaccine administered divided by the estimated population <1 y of age. Source: National Immunization Program, Ministry of Health, Brasilia, Brazil [31].

### Rotavirus Vaccination

Brazil's national immunization program operates within the Sistema Unico de Saúde (SUS), Brazil's publicly funded health-care system, to provide universal access to recommended vaccines [30]. The federal government purchases vaccines, which are distributed to state and local immunization programs and provided at no cost at public health facilities throughout the country. In April 2006, a rotavirus vaccine (Rotarix) was introduced into Brazil's national immunization program. Vaccination is recommended at 2 and 4 mo of age. The first dose can be administered at 6–14 wk, and the second dose at 14–24 wk of age.

Rotavirus vaccination coverage estimates for 2007 through 2009 among children under 1 y of age were obtained for each region from the information department of the Ministry of Health [31]. Doses of rotavirus vaccine administered during the years 2007 through 2009, registered as a first or second dose, were recorded in a national electronic database by clinics providing immunization services. Coverage with two doses of oral rotavirus vaccine was estimated as the number of second doses administered divided by the population <1 y of age in the corresponding calendar year.

### Diarrhea Admissions and Death Data

Data on diarrhea deaths were obtained from the Mortality Information System (Sistema de Informações sobre Mortalidade [SIM]) of the Brazilian Ministry of Health, the national database of information collected from death certificates [32]. This system is estimated to capture 90% of all deaths that occur in Brazil, with lower percentages (∼80%) in the North and Northeast regions. Capture of neonatal deaths is lower, but neonatal deaths account for a small proportion (∼3%–4%) of diarrhea deaths in Latin America [1]. Data on admissions were obtained from the electronic Hospital Information System (Sistema de Informações Hospitalares [SIH]) of SUS [32]. This system includes information on all hospital admissions authorized for payment by SUS, which covers approximately 70% of hospitalizations in Brazil. The hospitalization data are from public hospitals and some private hospitals that are paid by the government to care for patients. No changes occurred during the study period in terms of the number of hospitals that reported to this system. Both the SIM and SIH use the International Classification of Diseases (ICD-10) for causes of death (SIM) or admission diagnosis (SIH). In this analysis, we included deaths and admissions with the principal cause coded as diarrheal disease of any cause (ICD-10 codes: A00–A03, A04, A05, A06.0–A06.3, A06.9, A07.0–A07.2, A07.9, A08–A09) among children younger than 5 y from January 2002 through December 2009.

### Statistical Analysis

Rates of all-cause diarrhea-related deaths and admissions among children younger than 5 y of age were examined before (2002 through 2005) and after (2007 through 2009) introduction of rotavirus vaccination using an interrupted time-series analysis. We excluded data from 2006, the year in which the vaccine was introduced. These rates were calculated from SIM and SIH figures for diarrhea-related events per month and population, using annual projections from the 2000 census [33].

For the regression analysis, a generalized linear model was fit to the time-series data, assuming that the diarrhea deaths and admissions were Poisson distributed. The standard error of the rate ratio was scaled to the Pearson chi-squared statistic divided by the residual degrees of freedom to account for over-dispersion of the monthly counts in the outcome data for all models [34]. We first computed monthly rates of diarrhea-related deaths and admissions “expected” to occur in the absence of a rotavirus vaccination program by fitting the model to pre-vaccine data (2002 through 2005). We adjusted for seasonality by including an indicator variable for each calendar month and for secular trends by including calendar year in the model. The sequential year term captured the linear slope of decreasing secular trend; we assumed that this linear trend continued into the vaccine era (2007 through 2009). This model based on pre-vaccine data (including a constant and terms for month and year) was used to estimate expected values in the vaccine era. We then compared the absolute number of diarrhea-related deaths and admissions observed in the vaccine era with those expected in the absence of vaccination, as computed by the model, to assess the potential impact of vaccination. Finally, we calculated the rate ratio of diarrhea deaths and admissions in the vaccine era with the inclusion of an indicator variable for the period after rotavirus vaccine introduction; again, we controlled for seasonal and secular trends.

We investigated changes in rates of diarrhea deaths and admissions by age groups (under 1 y, 1 to <2 y, 2 to 4 y) because vaccine coverage during the early years of an immunization program and disease rates vary substantially by age. Separate models were also fitted for each region of Brazil in order to investigate differential impact of the vaccine program and to allow for different seasonality of rotavirus and secular trends. Results are presented as percent decline (1 − rate ratio) and 95% confidence interval (CI). Analyses were done with Stata (version 11).

## Results

### Vaccination Coverage

In 2007, when an estimated 80% of children younger than 1 y of age received two doses of rotavirus vaccine, only 47% of those 1 to <2 y of age, and none of those 2 to 4 y of age had been completely immunized. By 2009, an estimated 84% of children younger than 1 y of age (Table 1) 81% of children 1 to <2 y of age, and 36% of children 2 to 4 y of age had received two doses of rotavirus vaccine.

### Diarrhea-Related Deaths

**Figure 1:**
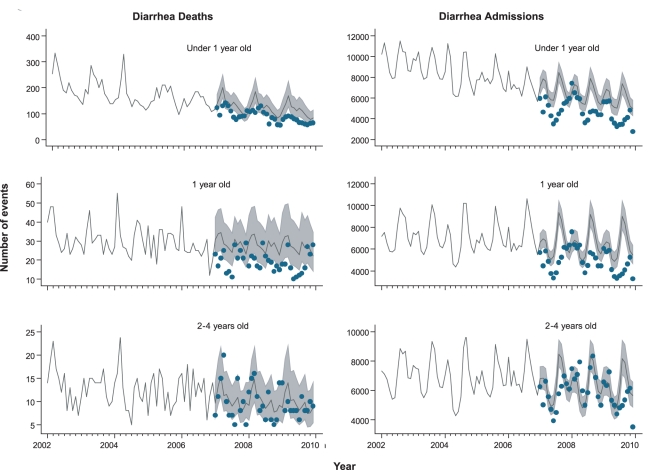
Trends in childhood diarrhea deaths and admissions in Brazil. Each analysis examines trends from 2002–2009, by age, including comparison of observed events (blue dots) after rotavirus vaccination (2007 to 2009) in Brazil with expected events (solid line) and 95% CIs (gray shaded area) in the absence of vaccination. Expected number of events and 95% CIs are based on predictions from regression models fitted to historic data from each region (2002 to 2005).

**Figure 2:**
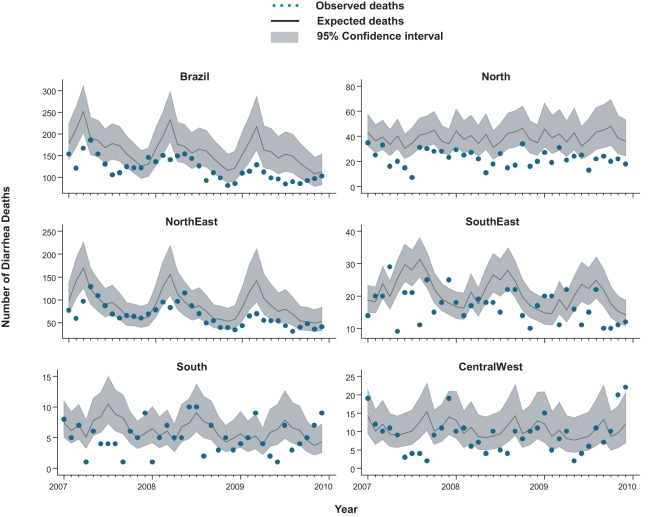
Impact of rotavirus vaccination on monthly events of childhood diarrhea deaths in Brazil by region. Each analysis compares the monthly observed events among children under 5 y after rotavirus vaccination (2007 to 2009) with expected events in the absence of vaccination, by region. Expected number of events and 95% CIs are based on predictions from regression models fitted to historic data from each region (2002 to 2005).

From 2002 through 2005, an annual median of 2,700 diarrhea-related deaths occurred among children younger than 5 y at an average annual rate of 16 deaths per 100,000, accounting for 14% of the 19,000 post-neonatal deaths (i.e., 30 d to 5 y of age) in Brazil each year. Prior to rotavirus vaccine introduction, there was a trend of declining diarrhea-related mortality among children younger than 1 y (relative reduction [RR] = 0.87/y; 95% CI 0.83–0.94; *p<*0.001), 1 to <2 y of age (RR = 0.96/y; 95% CI 0.91–1.02; *p = *0.23) and 2 to 4 y of age (RR* = *0.93/y; 95% CI 0.87–1.00; *p = *0.06) (Figure 1). Diarrhea mortality rates and seasonality differed significantly by region (Figure 2). A majority (73%) of diarrhea-related deaths occurred in the North and Northeast, where mortality rates were four to five times higher than those in the Central-West, South, and Southeast.

Compared to expected rates based on pre-vaccine trends, diarrhea-associated mortality among children younger than 5 y of age was 22% (95% CI 6%–44%) lower during the three years after rotavirus vaccination (2007 through 2009; Table 2). Additionally, in the month-to-month comparison of observed versus expected diarrhea mortality, a decline occurred during most months of the post-vaccination period (Figure 2), with most prominent reductions during January through March. Similar declines in diarrhea deaths among children younger than 5 y of age were observed during each post-vaccination year, with a 21% reduction (95% CI 6%–34%) in 2007, a 24% reduction (95% CI 7%–38%) in 2008, and a 33% reduction (95% CI 14%–47%) in 2009.

**Table 2:** Post-vaccination changes in numbers of diarrhea-related deaths and mortality rates among children younger than 5 y by December 2009 in Brazil.

Region and Age Group	Annual Number of Diarrhea Deaths Post-Vaccination (2007–2009)		Annual Death Rate (per 100,000) Post-Vaccination (2007–2009)		Percent Decline in Death Rates (95% CI)^c^
	Observed^a^	Expected^b^	Observed^a^	Expected^b^	
**All regions**					
<1 y	1,086	1,240	35	48	22 (6 to 35)
1 y	232	280	7	11	28 (6 to 45)
2–4 y	116	100	1	1	4 (30 to 29)
Total	1,435	1,610	9	12	22 (6 to 44)
**North**					
<1 y	194	276	61	88	25 (1 to 44)
1 y	57	180	18	56	61 (36 to 76)
2–4 y	22	48	2	4	61 (25 to 80)
Total	272	468	17	29	38 (21 to 51)
**Northeast**					
<1 y	624	864	61	86	20 (−5 to 40)
1 y	105	132	10	13	11 (−29 to 38)
2–4 y	59	60	2	2	−34 (−99 to 10)
Total	788	1,056	15	21	17 (−8 to 36)
**Southeast**					
<1 y	158	204	14	18	24 (4 to 41)
1 y	28	24	2	2	0 (−67 to 41)
2–4 y	17	12	0	0	−12 (−172 to 53)
Total	204	252	3	4	19 (−1 to 35)
**South**					
<1 y	42	60	12	17	33 (1 to 55)
1 y	12	12	3	2	11 (−133 to 66)
2–4 y	6	0	1	0	−63 (−694 to 62)
Total	61	72	3	4	26 (−8 to 50)
**Central-West**					
<1 y	68	72	29	32	11 (−38 to 43)
1 y	30	48	13	19	47 (−9 to 74)
2–4 y	11	12	2	1	37 (−85 to 72)
Total	110	132	9	11	22 (−15 to 48)

a Observed number of deaths are annual means for 2007–2009; observed rates are estimated annual rates for 2007–2009 from the regression model.

b Expected in the absence of vaccination on the basis of 2002–2005 data, adjusting for seasonality and secular trends.

c Calculated as 1 − RR of diarrhea deaths post-vaccine compared to the pre-vaccine era from Poisson regression models, adjusting for seasonality and secular trends.

**Figure 3:**
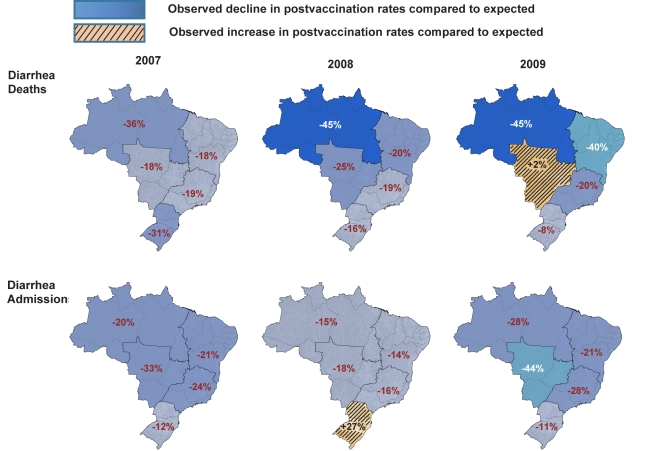
Change in diarrhea mortality and admission rates after vaccination by year and region. The maps depict the percent change in observed rates of diarrhea mortality and admission after rotavirus vaccination in Brazil compared to expected rates without a vaccination program, by year and region. Expected numbers of events are based on predictions from regression models fitted to historic data from each region (2002 to 2005). The percent declines are computed as one minus the RR of diarrhea events post-vaccine compared to the pre-vaccine era from Poisson regression models, adjusting for seasonality and secular trends.

Among all children younger than 5 y of age, observed mortality rates post-vaccination declined significantly in all regions, with largest absolute reductions occurring in high mortality regions of North and Northeast Brazil (Table 2). Among children younger than 1 y of age, observed rates were significantly lower than expected in all regions. Among 1- to <2-y-old children, the absolute declines in diarrhea-associated mortality in the North accounted for most of the overall declines in this age group. During each post-vaccination year, diarrhea-related mortality among children younger than 5 y of age was lower than expected in all but one region of Brazil (Figure 3). While a decline in mortality was not observed in the Central-West region (−2%; 95% CI −74% to 41%) in 2009 (Figure 3), overall diarrhea mortality during 2007–2009 for this region was non-significantly lower (22% decline; 95% CI −15% to 48%) than expected.

In total, during 2007–2009, approximately 1,500 fewer diarrhea-related deaths were observed in Brazil compared to that expected without a vaccine program among children under 5 y of age.

### Diarrhea-Related Admissions

During the pre-vaccine period, there were a total of ∼1,091,000 hospital admissions for diarrhea from all causes reported through the SUS among children younger than 5 y, giving an average annual rate of 1,593 per 100,000. Similar to diarrhea-related deaths, significant declining trends prior to vaccine introduction were also observed in diarrhea-related admissions among children younger than 1 y of age (RR* = *0.93/y; 95% CI 0.90–0.97; *p<*0.001), but not in children 1 to <2 y of age (RR* = *0.99/y; 95% CI 0.94–1.04; p* = *0.66) or 2 to 4 y of age (RR* = *0.98/y; 95% CI 0.94–1.03; *p = *0.59) (Figure 1). Unlike diarrhea-related deaths, which were mostly concentrated in the poorest North and Northeast regions, diarrhea-related hospital admissions occurred throughout Brazil. However, the highest expected diarrhea admission rates (per 100,000) were in the less developed North (2,915) and Northeast (2,070) and the lowest rates in the more developed Southeast (666) and South (892) regions of Brazil (Table 3).

**Table 3:** Changes in number and rates of hospital admissions for diarrhea due to all causes among children <5 y by December, 2009, Brazil.

Region and Age Group	Annual Number of Diarrhea Admissions Post-Vaccination		Annual Diarrhea Admission Rate (per 100,000) Post-Vaccination		Percent Decline in Admission Rates (95% CI)^c^
	Observed^a^	Expected^b^	Observed^a^	Expected^b^	
**All regions**					
<1 y	59,452	76,788	1,840	2,477	25 (14 to 34)
1 y	71,088	78,384	1,886	2,487	21 (7 to 33)
2–4 y	56,933	76,200	722	774	7 (−7 to 19)
Total	187,472	229,956	1,165	1,429	17 (5 to 27)
**North**					
<1 y	11,682	16,212	3,679	5,107	23 (10 to 34)
1 y	13,375	17,856	4,168	5,565	17 (1 to 30)
2–4 y	12,539	13,812	1,258	1,386	7 (−7 to 19)
Total	37,595	47,664	2,300	2,915	16 (3 to 27)
**Northeast**					
<1 y	26,240	36,324	2,584	3,578	27 (14 to 38)
1 y	27,097	36,708	2,661	3,605	23 (9 to 34)
2–4 y	32,987	34,344	1,064	1,108	6 (−9 to 19)
Total	86,325	106,284	1,681	2,070	19 (5 to 30)
**Southeast**					
<1 y	9,929	14,160	852	1,216	29 (14 to 42)
1 y	9,383	12,960	782	1,079	26 (5 to 42)
2–4 y	12,571	14,016	331	369	8 (−11 to 24)
Total	31,883	41,184	517	666	21 (3 to 36)
**South**					
<1 y	4,931	5,040	1,366	1,398	9 (−11 to 25)
1 y	5,140	5,148	1,374	1,378	2 (−27 to 24)
2–4 y	7,586	7,320	623	601	−6 (−37 to −17)
Total	17,657	17,412	904	892	0 (−24 to 20)
**Central-West**					
<1 y	4,151	5,784	1,759	2,451	24 (1 to 42)
1 y	4,456	6,984	1,871	2,938	30 (3 to 49)
2–4 y	5,404	7,764	743	1,069	31 (5 to 44)
Total	14,012	20,412	1,166	1,699	26 (4 to 44)

a Observed number of hospital admissions are annual means for 2007–2009; observed rates are estimated annual rates for 2007–2009 from the regression model.

b Expected in the absence of vaccination on the basis of 2002–2005 data, adjusting for seasonality and secular trends.

c Calculated as 1 − RR of diarrhea deaths post-vaccine compared to the pre-vaccine era from Poisson regression models, adjusting for seasonality and secular trends.

**Figure 4:**
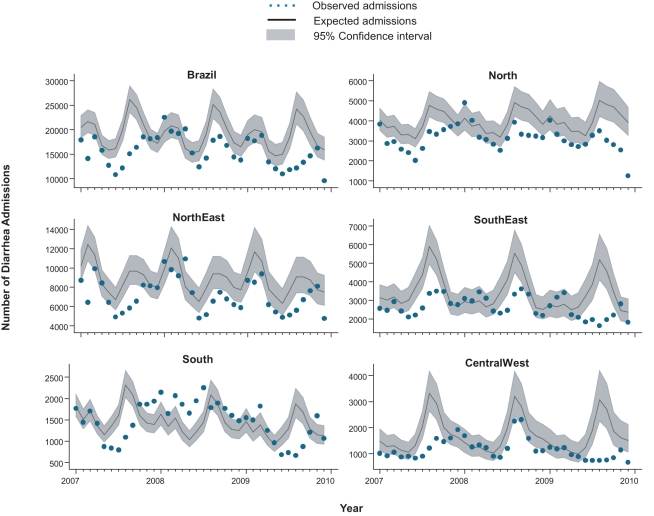
Impact of rotavirus vaccination on monthly events of diarrhea admissions in Brazil by region. Each analysis compares the monthly observed events among children under 5 y after rotavirus vaccination (2007 to 2009) with expected events in the absence of vaccination, by region. Expected number of events and 95% CIs are based on predictions from regression models fitted to historic data from each region (2002 to 2005).

During the three years following vaccine introduction, the observed annual hospital admission rates for diarrhea among children younger than 1 y and children 1 to <2 y of age were 637 and 601 per 100,000 lower than the expected rates without vaccination (Table 3), reductions of 25% (95% CI 14%–34%) and 21% (95% CI 7%–33%; *p<*0.001), respectively. Reduction in diarrhea-related hospital admissions was most prominent during May to October (Figure 4). Countrywide observed diarrhea-related hospital admissions among children younger than 5 y of age were lower than expected during each post-vaccination year, with a 21% reduction (95% CI 9%–31%) in 2007, an 11% reduction (95% CI −5% to 24%) in 2008, and a 24% reduction (95% CI 8%–37%) in 2009. Compared to expected rates, significantly lower diarrhea-related hospital admission rates were observed in four out of five regions (Table 3). In the South, diarrhea-related hospital admissions were 12% (95% CI −8% to 29%) lower than expected in 2007 and 11% (95% CI −19% to 33%) lower than expected in 2009, but above the expected by 27% (95% CI 0%–62%) during 2008 (Figure 3).

Overall, during 2007–2009, a 17% (95% CI 5%–27%) reduction, or approximately 130,000 fewer diarrhea-related hospital admissions (∼42,480 per year), was observed compared to the number of admissions expected in the absence of vaccination among children under 5 y of age in Brazil.

## Discussion

The introduction of a single-strain rotavirus vaccine in the childhood immunization program in Brazil was associated with a significant nationwide decline in diarrhea-related deaths and hospital admissions among children younger than 5 y of age. Reductions in mortality are consistent with results from Mexico, where the single-strain rotavirus vaccine was introduced in the same year (2006) [24]. However, Brazil is one of several countries in which substantial reductions in hospital admissions due to diarrhea have been shown following introduction of rotavirus vaccination into the national immunization program [19],[20]. Prevention of rotavirus-related admissions is particularly important for middle- and high-income countries in which nonfatal rotavirus diarrhea is a common cause of childhood morbidity. The results from Brazil suggest protection of rotavirus vaccination against both diarrhea deaths and diarrhea-related hospital admissions, adding to the strength of evidence supporting investment in rotavirus vaccination to curtail the 1.3 million deaths and 9 million hospital admissions related to diarrhea that occur annually worldwide.

In Brazil, we observed a reduction of approximately 40,000 admissions per year (i.e., nearly one in five diarrhea-related hospital admissions averted) among children under 5 y of age during 2007–2009. Declines in diarrhea-related hospital admissions were substantial both in the more developed South and Southeast regions, as well as in the poorest regions of Brazil (North and Northeast), where socioeconomic and health indicators approximate those of less developed countries. Because the baseline burden of diarrhea is three to five times greater in the North and Northeast than in other regions of Brazil, the absolute reduction in diarrhea-related deaths and diarrhea-related hospital admissions was much greater in this population. Lower rotavirus vaccine efficacy and immunogenicity have been observed in impoverished populations of Latin America, Asia, and Africa that have the highest risk of severe disease [11],[13],[18]. Significant reductions in diarrhea-associated mortality in the North of Brazil suggest that children living in areas with limited access to health care who are at highest risk of dying from diarrheal illnesses benefitted from vaccination.

Several pertinent findings support rotavirus vaccination as the most likely explanation for the reduction in diarrhea-related deaths and admissions. First, our estimates of decline were adjusted for seasonal fluctuations and secular declines in trends of diarrhea deaths and admissions. Second, the decline among vaccinated age groups was sustained on a national level for three full years after vaccine introduction. Third, the reduction in diarrhea-related hospital admissions was larger in children aged 1 y or less, who had high rates of vaccination, than in those 2 to 4 y of age, who were not eligible for rotavirus immunization during the study period.

Lastly, seasonal blunting in mortality and admissions was noted in several regions. Seasonality in rotavirus disease in Brazil varies by region and study [35]–[37]. In the South, Central-West, and Southeast regions of Brazil, previous surveillance indicated that seasonal peaks in rotavirus admissions occur during May to October, although year-round detection of the virus has also been reported [35],[36]. Our observation of the largest declines in diarrhea admissions during these months of peak rotavirus activity supports the contention that vaccination might have had a causal role. Data are sparse on the seasonality of rotavirus in the North and Northeast regions of Brazil, although one study suggests year-round detection of rotavirus [37], which is consistent with our finding of year-round decreases in diarrhea mortality in these regions.

We were intrigued by the increase in diarrhea-related hospital admissions during 2008 in the South despite high vaccination coverage, which contributed to an overall lower decline in diarrhea-related hospital admissions nationally during the same year. In 2007 and 2008, G2P[4] was the predominant rotavirus strain circulating in Brazil, a strain fully heterotypic to the G1P[8] vaccine used in Brazil, prompting a debate about whether vaccine pressure contributed to its emergence [38]–[40]. While emergence of this strain might explain the higher diarrhea-related hospital admission rates in the South during 2008, we think this is unlikely because the single-strain rotavirus vaccine provided good heterotypic cross-protection against G2P[4] strains in Brazil [26], and a similar G2P[4] strain predominated in the South as well as in other regions that had sustained declines in diarrhea-related disease. The possibility also exists that an accumulation of older susceptible children in 2008 who were unexposed to rotavirus during 2007 because of the large declines in rotavirus disease during that year might have contributed to an increase in the intensity of transmission during 2008 [41]. However, we suspect this is also unlikely to fully explain the increase in the South because similar increases should have been observed across Brazil. One possible explanation for greater than expected diarrhea-related hospital admissions in 2008 in the South of Brazil is circulation of other enteric pathogens causing a regional epidemic of diarrhea.

Several factors could affect the interpretation of our findings, primarily relating to the ecological nature of our study. Ecological studies can be informative but have several shortcomings. Because these studies do not link an exposure to an outcome at an individual level, they may be prone to ecological fallacy (i.e., those unexposed get the disease). Confounding biases such as seasonal periodicity of disease, secular declines in trends, contribution of simultaneously implemented interventions, and changes in coding and health-care treatment patterns cannot be directly assessed with these studies because of the absence of a control group [42]. Although we adjusted for seasonality and secular declines in diarrhea mortality and admissions, we cannot be fully confident that changes in coding or treatment practices did not occur during the study period. While we do not have evidence suggesting that reporting of diarrhea deaths changed over the study period, a decrease in reporting of deaths after rotavirus vaccine introduction could be misinterpreted as vaccine effectiveness. However, any changes in reporting would likely have affected all age groups, and led to decline of mortality or admissions in both older and younger children. The persistent reductions in diarrhea deaths and diarrhea-related hospital admissions across all regions, and the gradient in observed declines, with the lowest reduction in the oldest children, who were not immunized with the rotavirus vaccine, are consistent with vaccine-associated effects. Programmatic realities are such that diagnostic testing is typically not done for etiologic confirmation of diarrhea. Thus, we used all-cause diarrhea codes to assess vaccine impact. Although this may provide less precision with regard to vaccine effect on rotavirus disease, we suspect that measuring impact on all-cause diarrhea is more valuable to decision makers and the public health community because it provides an estimate of the preventable fraction of diarrhea deaths and admissions attributable to rotavirus.

In summary, this time-series analysis provides evidence of substantial reductions following the introduction of rotavirus vaccination of both diarrhea-related deaths and diarrhea-related hospital admissions from a large middle-income country in the Americas with both developing and developed regions. These findings have important global health policy implications. In low-income countries, the main impetus for introduction of rotavirus vaccines has been the potential to prevent rotavirus deaths: the consistency in findings of a sustained reduction in diarrhea mortality after rotavirus vaccination in Mexico [24] and Brazil suggests that rotavirus vaccination is an important tool for reducing the global burden of diarrhea deaths. In middle-income countries that are not eligible for financial support from donors, the potential reductions in diarrhea-related hospital admissions and other health-care costs will be important for cost-effectiveness considerations to justify the purchase of these relatively expensive vaccines. The reductions in diarrhea-related hospital admissions observed in Brazil, especially in low-mortality and higher income regions, are also relevant for decisions in higher income countries that have not yet introduced rotavirus vaccines into their routine immunization programs.

## Abbreviations

CI: confidence interval
HDI: human development index
RR: relative reduction
SIH: Hospital Information System (Sistema de Informações Hospitalares)
SIM: Mortality Information System (Sistema de Informações sobre Mortalidade)
SUS: Sistema Unico de Saúde
